# Whole-Genome Sequences of an Abortive Bacillus safensis Strain Isolated from a Mare’s Uterus

**DOI:** 10.1128/MRA.00342-20

**Published:** 2020-05-14

**Authors:** Sara V. Little, Andrew E. Hillhouse, Sara D. Lawhon

**Affiliations:** a Department of Veterinary Pathobiology, College of Veterinary Medicine & Biomedical Sciences, Texas A&M University, College Station, Texas, USA; b Texas A&M Institute for Genome Sciences and Society, College of Veterinary Medicine & Biomedical Sciences, Texas A&M University, College Station, Texas, USA; University of Maryland School of Medicine

## Abstract

This is a report of two Bacillus safensis genomes sequenced from separate cultures isolated from the uterus of a 16-year-old Westphalian mare that aborted a dead fetus. This strain represents the first case of a *B. safensis*-associated equine abortion and the first case of infection caused by this bacterium.

## ANNOUNCEMENT

This isolate came from a case described by Kelley et al. of a 16-year-old Westphalian (breed) mare from Texas that aborted a dead fetus at 7 months of gestation. Cultures from the mare’s uterus and the fetal lung tissue both showed heavy growth of a *Bacillus* sp., which was also seen within macrophages in the placental tissue, supporting this bacterium as the cause of the abortion (1). Matrix-assisted laser desorption ionization–time of flight (MALDI-TOF) mass spectrometry (Bruker Biotyper) was used to identify the bacterium as Bacillus pumilus, with MALDI-TOF previously shown to have the power to distinguish between closely related *Bacillus* species such as *B. pumilus* and B. safensis (2). A portion of the16S rRNA gene was amplified using the primers fD1mod (5′-AGAGTTTGATCYTGGYTYAG-3′) and 16S1RR-B (5′-CTTTACGCCCARTRAWTCCG-3′) as previously described (3). Two cultures from the mare’s uterus were provided for whole-genome sequencing and analysis, which allowed for the correct assignment of the species as *B. safensis*. This is a species first identified from spacecraft and assembly facility surfaces (4) that is currently being evaluated for biotechnological applications (5).

The isolates from the mare’s uterus were initially grown on Trypticase soy agar supplemented with 5% sheep blood and incubated at 35°C ± 2°C with 5% carbon dioxide added overnight. A freezer stock was made in brucella broth supplemented with 10% glycerol. The isolates were revived using the same culture method, and a single colony was used to inoculate Trypticase soy broth and incubated overnight under the conditions described above. For DNA extraction, 1-ml aliquots of each isolate were pelleted and subsequently lysed in a Qiagen TissueLyser using Macherey-Nagel bead tubes (type B) and lysis buffer from the NucleoMag tissue DNA kit. DNA isolation was performed using the manufacturer’s protocol (Macherey-Nagel). The quality of genomic DNA was verified on a genomic DNA TapeStation run (Agilent) prior to sequencing.

For all of the following programs, default parameters were used except where otherwise specified.

Illumina libraries were prepared following the manufacturer’s protocol for the Illumina Nextera DNA Flex library preparation kit and sequenced on an Illumina MiSeq V3 2 × 300 kit. All data were uploaded to Illumina’s cloud-based resource, BaseSpace, for run monitoring, FASTQ generation, demultiplexing, and adapter trimming. The sequencing output of paired-end read sets contained approximately 2.4 and 2.0 million reads of 301 bp for the two isolates, respectively, resulting in over 150× coverage.

Reads were assembled using SPAdes version 3.13.0 (6) using the parameter –careful. The assemblies were 57 and 65 contigs in total, consisting of a total of 3,815,098 and 3,718,887 bp and *N*_50_ contig lengths of 542,045 and 542,046, respectively, with an approximate GC content of 40%. The longest contigs were 883,269 and 833,136 bp, respectively. Completeness was analyzed using Benchmarking Universal Single-Copy Orthologs (BUSCO) version 3 (7, 8) and the *Firmicutes* OrthoDB version 9 data set. Both assemblies scored 100% completeness.

The two uterus isolates were submitted for NCBI average nucleotide identity (ANI) analysis (9). The ANI analysis found 97% identity to *B. safensis* and 91% identity to *B. pumilus*, determining the species as *B. safensis* and highlighting the importance of using whole-genome sequencing for confirming species identification.

### Data availability.

The two whole-genome sequences were deposited in GenBank under BioProject number PRJNA566412 and accession numbers WCHP00000000 and WCHQ00000000. The raw reads are available under SRA accession numbers SRX6874088 and SRX6874087, respectively (Illumina FASTQ). This announcement represents the first version of these genomes.
